# Abiraterone Acetate Affects Gene Expression Profile in a Human Male Neuronal Cell Line: Potential Mechanism for Cognitive Deficits with Prostate Cancer Therapy

**DOI:** 10.3390/life16071184

**Published:** 2026-07-16

**Authors:** Shelly Gulkarov, Allison B. Reiss, Ankita Srivastava, Jasper Lim-Goyette, Heather A. Renna, Andrew Laccetti, Aaron E. Katz

**Affiliations:** 1 Department of Foundations of Medicine, NYU Grossman Long Island School of Medicine, Mineola, NY 11501, USA; shelly.gulkarov@nyulangone.org (S.G.); ankita.srivastava@nyulangone.org (A.S.); jlimgoyette@gmail.com (J.L.-G.); heather.renna@nyulangone.org (H.A.R.); 2 Department of Medicine, NYU Grossman Long Island School of Medicine, Mineola, NY 11501, USA; andrew.laccetti@nyulangone.org; 3 Department of Urology, NYU Grossman Long Island School of Medicine, Mineola, NY 11501, USA; aaron.katz@nyulangone.org

**Keywords:** abiraterone acetate, neuron, prostate cancer, mitochondrial dysfunction, low-density lipoprotein receptor-associated protein-1, synaptophysin, androgen

## Abstract

Background and Objectives: Cornerstone therapies for metastatic prostate cancer include androgen deprivation and androgen receptor pathway inhibition, but cognitive impairment is a recognized, life-altering potential adverse effect of this treatment. Abiraterone acetate (AA), an androgen receptor pathway and CYP17A1 inhibitor, suppresses androgen synthesis and may contribute to cognitive changes. This cell culture-based study uses the BE(2)M17 human male neuroblastoma model to investigate AA-induced alterations in gene and protein expression that may underlie cognitive decline, laying the foundation for a mechanistic investigation aimed at identifying molecular targets to mitigate cognitive impairment in men with prostate cancer receiving androgen-directed therapies. Materials and Methods: BE(2)M17 cells were pretreated for 12 h with dihydrotestosterone (DHT; 5 nM) or vehicle control, then exposed to AA (0, 5, 10 µM, 24 h). RNA and protein were analyzed by qRT-PCR and Western blot for markers of amyloid processing, neuronal health, and mitochondrial function. Results: AA significantly altered multiple neurobiological markers. BACE1 mRNA increased in DHT + 10 µM AA compared to control and DHT alone (*p* = 0.0416 and *p* = 0.0118). However, BACE1 protein decreased in 10 µM AA + DHT versus DHT alone (*p* = 0.0132). Immunoblot revealed reduced amyloid precursor protein (APP) in 10 µM AA versus control (*p* = 0.0057) and 10 µM versus 5 µM (*p* = 0.0263). APP was reduced in 10 µM AA + DHT versus control (*p* = 0.0015) and versus DHT alone (*p* = 0.0467). LRP1 and BDNF were significantly reduced with 10 µM AA versus 5 µM AA (*p* = 0.0092 and *p* = 0.0081), while synaptophysin decreased in 10 µM AA + DHT versus DHT alone (*p* = 0.0049). BDNF also declined in 10 µM AA + DHT compared to 5 µM AA + DHT (*p* = 0.0301). PGC1α mRNA increased in AA + DHT versus DHT alone (*p* = 0.0332). MitoTracker analysis showed reduced fluorescence with 5 µM AA alone but increased fluorescence with 5 µM AA + DHT relative to control and DHT (*p* = 0.0019; *p* < 0.0001), while 10 µM AA + DHT reduced fluorescence compared to 5 µM AA + DHT (*p* = 0.0003). Conclusions: AA, alone or combined with DHT, disrupts key pathways involved in neuronal health, amyloid processing, and mitochondrial function. These findings suggest a potential mechanistic link between AA treatment and cognitive impairment.

## 1. Introduction

For men worldwide, prostate cancer is the second most common solid cancer and the fifth leading cause of cancer death [1]. Over 300,000 cases are diagnosed in the United States annually, resulting in approximately 35,000 cancer deaths [2]. Prostate cancer cells are fueled by androgens, including testosterone and dihydrotestosterone. The vast majority of untreated prostate cancer demonstrates sensitivity to androgen deprivation therapy (ADT) with or without androgen receptor pathway inhibitors (ARPIs), which is the mainstay systemic therapy for patients with advanced disease [3].

Abiraterone acetate (AA) reduces androgen production by selectively and irreversibly inhibiting CYP17A1, which includes 17-α-hydroxylase and 17,20-lyase [4]. CYP17A1 is located in the endoplasmic reticulum of the testis, adrenal glands, adipocytes, and prostate cancer cells, where it plays a significant role in the synthesis of steroid hormones including mineralocorticoids, cortisol, and androgens. Cortisol or androgen production is initiated when cholesterol is converted to pregnenolone, which is then converted to progesterone [5]. 17-α-hydroxylase converts progesterone into 17-hydroxyprogesterone. The inhibition of 17-α-hydroxylase by AA results in the blocking of conversion of 17-hydroxyprogesterone to dehydroepiandrosterone and androstenedione, the final precursors of testosterone formation. AA is an approved treatment for high-risk localized and metastatic prostate cancer, extending the duration of cancer control and overall survival compared to ADT monotherapy [6,7,8].

A number of studies have demonstrated a link between hormone deprivation and reduced cognitive function [9]. Pharmacologic blockade of androgen effects is connected to mood changes, diminished quality of life, and dementia [10,11]. Several studies propose plausible mechanisms to explain hormone-associated cognitive changes. Androgens (testosterone, dihydrotestosterone (DHT), dehydroepiandrosterone sulfate) activate androgen receptors localized at the neuronal plasma and mitochondrial membranes [12]. Testosterone also improves mitochondrial function and neurotransmission by activating ERα and Erβ, making its impact on mitochondrial function crucial [13]. A previous cell culture study found that AA inhibits proliferation and promotes apoptosis of prostate cancer cells through regulating mitophagy [14]. While there is currently no strong evidence that abiraterone’s mitophagy effect in prostate cancer directly translates to neurons, neurons are particularly vulnerable to impairment of bioenergetics stemming from excessive or dysregulated mitophagy [15]. Other potential mechanisms include neuroinflammation and blood–brain barrier (BBB) disturbances related to hormone depletion [16,17]. Neuroinflammation is thought to be mediated by microglial activation and cytokine release, while BBB integrity diminishes with disruption in tight junction structures and up-regulation of inflammatory markers.

Several androgen-independent pathways could explain the cognitive effects of AA. CYP17A1, present in both neurons and astrocytes, is responsible for forming the neuroprotective steroid DHEA, and its inhibition by AA could reduce DHEA and other neurosteroid levels. CYP17A1 inhibition may also damage the endoplasmic reticulum, inducing oxidative stress and the accumulation of reactive oxygen species [18,19]. In addition to depleting neurosteroid availability, in adult male rats, AA has been demonstrated to disrupt the mesocorticolimbic dopaminergic system, altering behavioral flexibility [20]. Lastly, inhibition of CYP17A1 lowers cortisol synthesis, triggering hypothalamic-pituitary-adrenal axis compensation that disrupts glucocorticoid-dependent synaptic plasticity pathways crucial for executive function and memory [21,22].

Some prostate cancer patients may avoid life-prolonging ADT and ARPIs due to intolerable neurocognitive symptoms including cognitive slowing, short-term memory impairment, and personality change. Pembroke et al. also demonstrate the association between psychosocial factors such as depression, anxiety, fatigue, and insomnia with cognitive problems in prostate cancer survivors receiving hormonal therapy [23]. In a meta-analysis with 12 randomized controlled trials totaling 13,524 patients, a significant risk of cognitive toxic effects and fatigue was noted in patients treated with second-generation hormone therapy including AA, with any grade of cognitive toxic effects reported in 2% to 8% of participants [24]. Another meta-analysis reported that AA is associated with a significant increase in cognitive impairment risk [25]. Discontinuation due to cognitive symptoms is underreported, with cognitive issues being subsumed into the broader category of toxicity issues [26].

In light of this well-substantiated link between neurocognitive symptoms and androgen inhibition, the present study explored the role of AA in neuronal health and mitochondrial function using a human neuronal cell culture model.

## 2. Materials and Methods

### 2.1. Cell Culture and AA Treatment

Male BE(2)M17 neuroblastoma cells were cultured in a 1:1 mixture of Eagle’s Minimum Essential Medium with non-essential amino acids and F12 medium containing 10% fetal bovine serum in a humidified cell culture incubator with 5% carbon dioxide. At 70–80% confluency, the cells were seeded at a density of 500,000 cells/well in multiwell six-well plates (Corning Inc., Corning, NY, USA) in complete EMEM/Ham’s F12 media. After 48 h; media was switched to 1:1 EMEM/Ham’s F12 complete with 10% charcoal-stripped FBS, 1% PenStrep, and 1% Glutamax. After 48 h, cells were incubated with 5 nM DHT for 12 h. After 12 h of DHT treatment, the cells were subsequently exposed to 0, 5, and 10 µM AA ± 5 nM DHT. Control vehicles used are DMSO for AA and methanol for DHT. DMSO is used as a control vehicle for the experimental groups without DHT, while DMSO + methanol is the control vehicle for the experimental groups with DHT. Experiments were performed after 24 h of AA treatment.

### 2.2. Real-Time PCR

Following AA treatment for 24 h, total RNA was isolated using Trizol reagent. RNA phase separation was performed using 1-Bromo-3-chloropropane (BCP), then precipitated with isopropanol, washed with ethanol, and resuspended in diethyl pyrocarbonate (DEPC) water. RNA concentrations were measured using a Thermo Scientific™ NanoDropT™ OneC Microvolume UV-Vis Spectrophotometer (Thermo Fisher Scientific Inc., Waltham, MA, USA). Complementary DNA (cDNA) was synthesized using reverse transcriptase reagents obtained from ThermoFisher Scientific as follows per sample: 25 μM MgCl_2_, 10× PCR buffer, 10 mM dNTP, oligodeoxyribonucleotide (Oligo dT), random hexamers, RNase A inhibitor, and reverse transcriptase Moloney Murine Leukemia Virus (MUL-V). 1 ug of RNA per sample was used for cDNA preparation. cDNA was then synthesized in an Eppendorf Mastercycler Nexus GX2 PCR machine with the following protocol: 5 min at 25 °C, 60 min at 42 °C, 5 min at 80 °C, hold at 4 °C. Samples were brought up to final volume using DEPC water.

The cDNA was used for quantitative real-time PCR analysis on a Light Cycler 480 system (Roche, Indianapolis, IN, USA) with the FastStart SYBR Green Reagents Kit according to instructions provided by the manufacturer. Gene expression levels were calculated by using the (2−ΔΔCt) method and normalized to the expression of the endogenous control, GAPDH. The list of primers used is provided in tabular form (Table 1).

### 2.3. Western Blotting

Protein samples were collected from whole cell lysates prepared by sonication in radioimmunoprecipitation assay (RIPA) lysis buffer (98% PBS, 1% Igepal, 0.5% sodium deoxycholate, 0.1% sodium dodecyl sulfate, supplemented with 10 μL per mL of protease inhibitor cocktail (Sigma-Aldrich, St. Louis, MO, USA)) after 24 h of AA treatment. The concentration of protein was determined using the BCA Protein Assay Kit (Pierce Biotechnology Inc., Rockford, IL, USA). 15 µg of each protein sample was loaded and separated by 8–12% SDS-polyacrylamide gel electrophoresis (SDS-PAGE). Separated proteins were transferred to a nitrocellulose membrane. The membrane was then subsequently blocked with 5% milk in TBST for 2 h at room temperature and then incubated in primary antibodies at 4 °C overnight. After overnight incubation, the membrane was then incubated with secondary antibodies diluted 1:5000 in 2% BSA in TBS. The immunoreactive protein was detected using ECL Western blotting detection reagents (Thermo Scientific™ SuperSignal™ West Pico PLUS Chemiluminescent Substrate) and the Bio-Rad ChemiDoc Touch Imaging System. Loading in each lane was validated using β-actin as an internal loading control. Quantification of protein blots was performed using ImageJ software (version 1.54t).

### 2.4. MitoTracker Staining

Human male BE(2)M17 cells were stained with MitoTracker dye that stains active mitochondria in live cells. After 48 h, cells were incubated with 5 nM DHT for 12 h. After 12 h of DHT treatment, the cells were treated with 0, 5, and 10 µM AA ± 5 nM DHT. Following 24 h of AA treatment, cells were washed with Hanks’ Balanced Salt Solution (HBSS) and stained with 250 µM of MitoTracker (Thermo Fisher Scientific, M7512) for 20 min. Cells were washed with HBSS after 20 min of staining. Images were acquired at 20× magnification under a fluorescence microscope, and total cellular fluorescence was quantified using ImageJ.

### 2.5. Statistics

Results were reported as Mean ± SD. Statistical significance was analyzed by one-way ANOVA followed by Bonferroni’s multiple comparison test. Data were analyzed and graphed on GraphPad Prism software.(version 11.0.2). Fold change values between the control and treated groups were calculated. *p* values less than <0.05 were considered significant.

## 3. Results

### 3.1. Effects of AA on Genes Regulating Amyloid-β Formation in BE(2)M17 Cells

**Figure 1:**
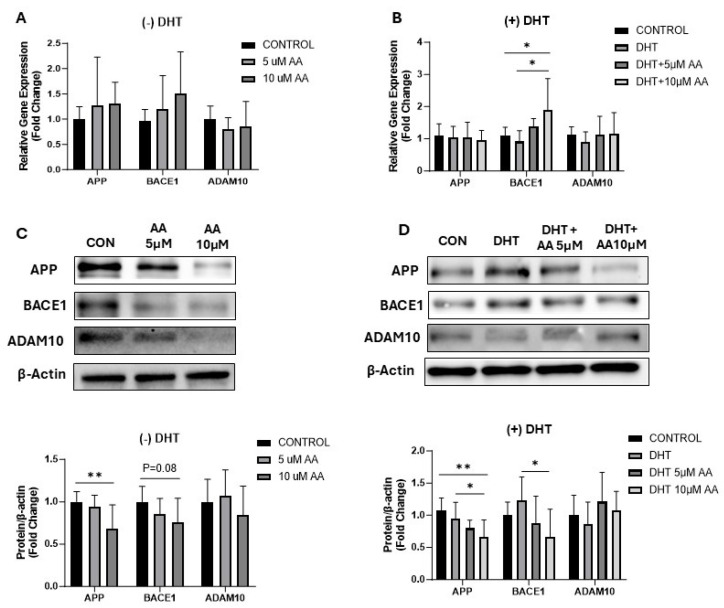
Effect elicited by AA on expression of genes involved in amyloid-β formation. (**A**,**B**) Real-time PCR analysis of APP, BACE1, and ADAM10 in BE(2)M17 cells treated with AA (0, 5, 10 µM) in the absence or presence of DHT (5 nM). GAPDH was used as an internal control (N = 9). (**C**,**D**) Western blot analysis of APP, BACE1, and ADAM10 in BE(2)M17 cells treated with AA (0, 5, 10 µM) in the absence or presence of DHT (5 nM). Densitometry of a representative blot was normalized to β-actin. Data were represented in fold difference. N = 9, * *p* < 0.05, ** *p* < 0.01, based on a one-way ANOVA followed by Bonferroni multi-comparison tests.

To explore the role of AA in regulating amyloid-β formation, we measured the expression of genes involved in amyloid-β formation such as APP, BACE1, and ADAM10 in BE(2)M17 cells at both the mRNA and protein levels following AA treatment in the absence and presence of DHT. Real-time PCR analysis showed that AA treatment in the absence of DHT did not change the mRNA levels of APP, BACE1, and ADAM10 at either AA concentration compared to the control group. However, treatment with AA at the 10 µM concentration in the presence of DHT significantly increased the mRNA level of BACE1 compared to the control and DHT conditions, while no changes were observed in APP or ADAM10 mRNA levels (Figure 1A,B).

Furthermore, Western blot analysis showed that AA treatment at the 10 µM concentration in the absence of DHT reduced the protein levels of APP and BACE1 compared to the control, whereas no change in ADAM10 protein levels was observed. Treatment with AA at the 10 µM concentration in the presence of DHT significantly reduced APP protein levels compared to both the control and DHT conditions, while BACE1 protein levels were reduced at 10 µM AA concentration compared to the DHT alone condition, and no change in ADAM10 protein levels was observed (Figure 1C,D).

### 3.2. Modulation of Expression of Genes Involved in Neuronal Health by AA

**Figure 2:**
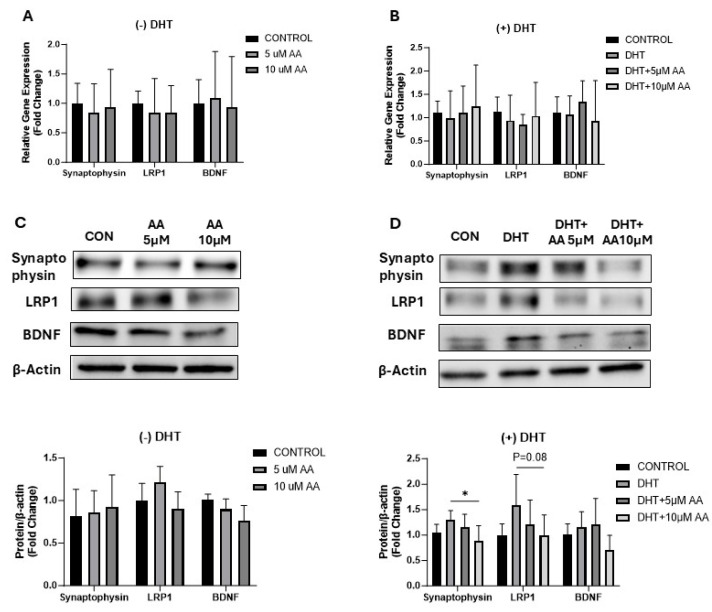
Effect of AA on genes contributing to neuronal health. (**A**,**B**) Real-time PCR analysis of synaptophysin, LRP1, and BDNF in BE(2)M17 cells after AA treatment (0, 5 µM and 10 µM) in the absence or presence of DHT (5 nM). GAPDH was used as an internal control (N = 9). (**C**,**D**) Western blot analysis of synaptophysin, LRP1, and BDNF in BE(2)M17 cells after AA treatment (0, 5 µM and 10 µM) in the absence or presence of DHT (5 nM). Densitometry of a representative blot was normalized to β-actin. Data were represented in fold difference. N = 9, * *p* < 0.05, based on a one-way ANOVA followed by Bonferroni multi-comparison tests.

To investigate the role of AA treatment in neuronal health, we quantified the expression of synaptophysin, BDNF, and low-density lipoprotein receptor-related protein 1 (LRP1) in BE(2)M17 cells in the absence and presence of DHT. Real-time PCR analysis showed that mRNA levels of synaptophysin, LRP1, and BDNF did not change with AA treatment either with or without DHT exposure compared to control or DHT conditions (Figure 2A,B). Furthermore, we performed Western blot analysis to determine protein levels and found that AA treatment did not alter the protein levels of synaptophysin, LRP1, or BDNF at any of the AA concentrations in the absence of DHT. However, AA treatment at the 10 µM concentration in the presence of DHT significantly reduced the protein levels of synaptophysin and LRP1 compared to DHT alone, while no change in BDNF protein levels was observed (Figure 2C,D).

### 3.3. Effects of AA on Genes Associated with Mitochondrial Function

**Figure 3:**
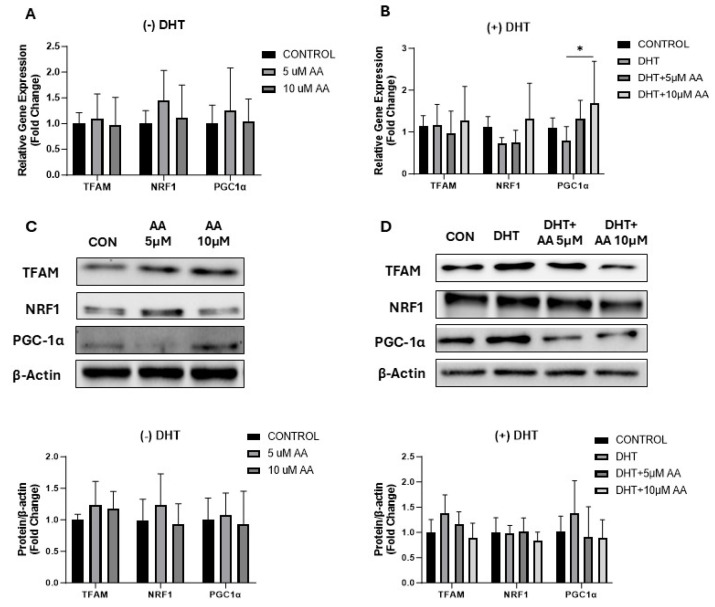
Effect of AA on genes involved in regulating mitochondrial biogenesis and functioning. (**A**,**B**) Real-time PCR analysis of TFAM, NRF1, and PGC1α mRNA in BE(2)M17 cells after AA treatment (0, 5 µM and 10 µM) in the absence or presence of DHT (5 nM). GAPDH was used as an internal control (N = 9). (**C**,**D**) Western blot analysis of TFAM, NRF1, and PGC1α in BE(2)M17 cells after AA treatment (0, 5 µM and 10 µM) in the absence or presence of DHT (5 nM). Densitometry of a representative blot was normalized to β-actin. Data were represented in fold difference. N = 9, * *p* < 0.05, based on a one-way ANOVA followed by Bonferroni multi-comparison tests.

To evaluate the influence of AA treatment on the expression of genes relevant to mitochondrial homeostasis, we measured the expression of TFAM, NRF1, and PGC1α at both the mRNA and protein levels in the presence and absence of DHT in BE2M17 cells. Real-time PCR analysis showed no change in TFAM, NRF1, and PGC1α mRNA levels after AA treatment in the absence of DHT. However, AA treatment at the 10 µM concentration in the presence of DHT significantly increased the mRNA levels of PGC1α as compared to DHT alone, while no change in mRNA levels of TFAM and NRF1 was detected (Figure 3A,B). Furthermore, we did not find any difference in the protein levels of TFAM, NRF1, or PGC1α following AA treatment in either the absence or presence of DHT (Figure 3C,D).

### 3.4. AA Treatment and Mitotracker Staining

**Figure 4:**
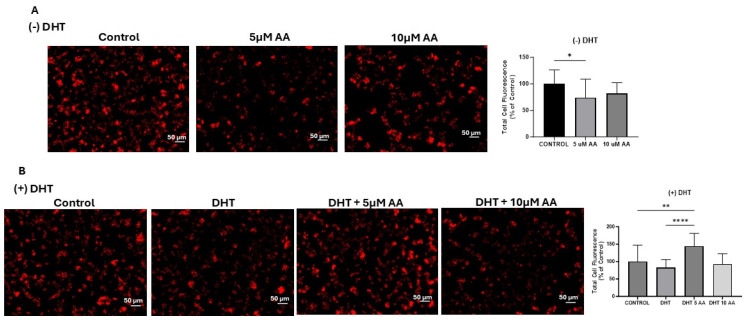
Effect of AA treatment on mitochondrial health and biogenesis. (**A**,**B**) Fluorescence microscopic images of live BE(2)M17 cells stained with 250 µM of MitoTracker after AA treatment (0, 5 µM and 10 µM) in the absence or presence of DHT (5 nM). Scale bar: 50 µM. Total cell fluorescence was measured using ImageJ software. * indicates *p* < 0.05, ** indicates *p* < 0.01, **** indicates *p* < 0.0001; based on one-way ANOVA followed by Bonferroni test for multiple comparisons.

To assess mitochondrial health and biogenesis, we stained BE(2)M17 cells with MitoTracker stain after AA treatment in the absence or presence of DHT. Following AA treatment, fluorescence images showed a reduction in active mitochondria at only the 5 µM concentration of AA as compared to control in the absence of DHT (Figure 4A). However, AA treatment in the presence of DHT significantly increased the active mitochondria at the 5 µM concentration of AA as compared to control and DHT-alone conditions (Figure 4B).

## 4. Discussion

In the present study, we evaluated whether AA exhibits neurotoxic effects on BE(2)M17 cells in the absence and presence of DHT by examining critical gene expression and mitochondrial health. Our data showed that exposure to AA significantly reduced APP protein levels, which is novel and yet to be described in available literature. While this reduction in APP may superficially appear neuroprotective in the context of amyloid biology, the functional implications are complex because APP participates in multiple cleavage pathways and generates products with divergent roles. Our results showed no significant change for ADAM10, which acts on APP in a non-amyloidogenic pathway. In addition, the significant decrease in amyloidogenic BACE1 at DHT + 10 µM AA compared to DHT alone, and the trend toward a decrease in BACE1 without DHT at 10 µM AA compared to the control, illustrates that AA may be shifting APP processing away from the amyloidogenic pathway, even if ADAM10 is unchanged [27]. However, this is paradoxically contradicted by an increase in BACE1 mRNA at DHT + 10 µM AA compared to the control. This suggests a compensatory transcriptional response despite reduced BACE1 protein, reflecting a dissociation between transcript and protein abundance due to post-transcriptional regulation that warrants further investigation [28]. Furthermore, APP has been shown to be beneficial for synaptic transmission and plasticity as exemplified in APP knockout murine models that display hippocampal synaptic plasticity impairment [29,30]. Therefore, the significant reduction of APP may not simply be explained by protection against amyloid pathology—rather, a complex mechanism involving a gene-protein dissociation suggests disruption of normal neuronal homeostasis in the context of androgen receptor inhibitors. In a recently reported ARACOG trial, cognitive impairment was the most common reason for switching from enzalutamide to darolutamide during the trial, demonstrating the importance of differential effects of ARPIs on cognitive function [31]. Although the exact mechanism behind the cognitive differences is not entirely understood, it is hypothesized to be due to the differences in BBB penetration between enzalutamide and darolutamide [32].

Our data also showed a decrease in neuroprotective protein levels with a decrease in LRP1 at 10 µM AA compared to 5 µM AA without DHT and trending towards significance at DHT + 10 µM AA compared to DHT alone. LRP1 has a dual role in the metabolism of APP, where it regulates receptor-mediated amyloid-β uptake and degradation in astrocytes and neurons [33]. As the primary receptor for amyloid-β clearance at the blood–brain barrier, it plays a crucial role in amyloid-β metabolism, facilitating both amyloid-β uptake and clearance [34]. These opposing roles of LRP1 in amyloid-β metabolism should be contextualized with the significant decrease in LRP1 at 10 µM AA compared to 5 µM AA without DHT and trending towards significance at DHT + 10 µM AA compared to DHT alone, respectively. While a decrease in LRP1 could imply less amyloid-β uptake in neurons, it could simultaneously impair amyloid-β clearance from the extracellular space. This dysregulation in amyloid-β uptake and clearance agrees with current literature that, although limited to retrospective studies that are associative and not causative, indicates a higher risk of Alzheimer’s disease and dementia in prostate cancer patients undergoing ADT [35,36]. While the exact mechanism is unclear, low testosterone levels have been correlated with an increase in amyloid-β in the brain and body fluid [37]. A previous study used antisense against LRP-1 mRNA and found that it decreased LRP-1 expression, reduced blood–brain barrier clearance of amyloid-β_42_, increased brain levels of amyloid-β_42_, and impaired learning ability and recognition in mice [38]. Overall, LRP-1 plays a crucial role in amyloid-β metabolism, and the significant decrease at 10 µM AA compared to 5 µM AA without DHT, and trend towards significance at DHT + 10 µM AA compared to DHT alone, may be a vital link to linking dysregulation in amyloid-β metabolism to known dementia risk associated with ADT. This link remains mechanistically unclear and should be further explored.

Other crucial neuroprotective proteins are synaptophysin and BDNF. Our study found a significant decrease in BDNF at 10 µM AA compared to 5 µM AA without DHT, and a significant decrease in BDNF and synaptophysin at DHT + 10 µM AA compared to DHT + 5 µM AA and DHT alone, respectively. BDNF is a neuropeptide that plays an important role in synaptic plasticity, while synaptophysin accelerates synaptic vesicle exocytosis and release of neurotransmitters, thereby facilitating signaling [39,40]. This decrease in two critical proteins for neuronal health and plasticity signifies an impairment of presynaptic vesicle function and synaptic plasticity that occurs in an androgen-dependent manner and could be a contributing factor to the cognitive symptoms observed in the low androgen state [41].

No significant changes were found in mitochondrial gene expression or protein levels. However, MitoTracker staining of live cells demonstrated significant changes with and without DHT, indicating alterations in mitochondrial health and biogenesis. Our results found a significant decrease in total cell fluorescence at 5 µM AA compared to the control without DHT, indicative of decreased mitochondrial health. No significant differences were found between 10 µM AA and 5 µM AA or the control. This is consistent with literature that shows testosterone deficiency worsens cognitive impairment in AD patients, such as a decrease in hippocampal mitochondrial function and mitochondrial biogenesis impairment in animal studies [42]. However, androgen signaling appears to modulate this inhibitory mitochondrial effect, as we found a significant increase at DHT + 5 µM AA compared to the control, DHT alone, and DHT + 10 µM AA. This paradoxical increase is only observed at the DHT + 5 µM AA dose and reverts at DHT + 10 µM AA and therefore appears to be dose-dependent. A similar study performed on LNCaP prostate cancer cells found that AA induced mitophagy and apoptosis in this cell type, indicating that AA can influence mitochondrial homeostasis and cell fate in an androgen-sensitive context [14]. To our knowledge, this is the first study of the effect of AA on mitochondrial biogenesis in neurons. Future studies are planned focusing on the effect of AA on mitochondrial biogenesis in neurons and prostate cancer cells in a co-culture model.

Although AA has limited blood–brain barrier penetration, it was selected because its potent suppression of systemic androgen synthesis may still alter brain-relevant signaling and downstream neuronal pathways implicated in cognition, independent of direct CNS drug exposure [43]. Further, AA is a widely used standard-of-care therapy for advanced prostate cancer, making its cognitive effects of high clinical relevance, possibly impacting a large patient population [44,45]. A case report from Switzerland found that a combination of abiraterone and ADT conferred sustained cerebral tumor response in a man with prostate cancer metastases to the brain, suggesting that AA has at least some CNS access [46].

Several limitations should be acknowledged. As a cell culture study, this work cannot determine the in vivo impact of ADT or the testosterone-depleted state on neuronal biology. In addition, AA’s effects on neural cells were not directly evaluated, and its actions are likely to involve mechanisms beyond androgen suppression alone. Our findings did not directly assess the mechanisms that cause the changes in mitochondrial biogenesis and neuronal health/pathways, and although these changes can be hypothesized to be affected by androgen-independent mechanisms such as CYP17A1 inhibition and neurosteroid depletion, our study design did not directly test this. This in vitro study cannot fully recapitulate the complexity of crosstalk among microglial activation, BBB dynamics, and systemic endocrine feedback loops. To address these limitations, we are initiating a prospective human study that will combine serum analyses, brain MRI, and cognitive testing in patients receiving ADT with or without ARPIs.

The present study was designed to establish that AA alters gene expression in neural cells in a pattern consistent with cognitive dysfunction, rather than to delineate the causal role of individual genes or pathways. Accordingly, we did not perform targeted mechanistic intervention experiments, such as siRNA-mediated knockdown, pharmacologic inhibition, or miRNA mimic gain-of-function approaches, to functionally validate specific mitochondrial fission/fusion or synaptic plasticity candidates. Future studies will prioritize these functional analyses in complementary neural models to clarify the extent to which these pathways mediate abiraterone acetate–associated cognitive decline.

In this study, we employed the male human neuroblastoma–derived BE(2)-M17 neural cell line as our primary model to investigate AA–associated cognitive mechanisms. This model was selected because it is widely used in the neurodegeneration and neurotoxicity literature and provides a neuronal context suitable for probing mitochondrial dynamics, synaptic plasticity–related pathways, and other processes implicated in cognitive impairment [47,48]. Furthermore, because BE(2)-M17 cells originate from a male patient, they provide a reliable, active XY model for investigating androgen-induced transcriptional changes. This minimizes the confounding effects of X chromosome dosage and female-specific epigenetic compensation that may be present in XX-derived lines such as SH-SY5Y, thereby enabling more precise assessment of androgen-sensitive pathways in a male genetic context [49].

Within the scope of the present work, our aim was to define AA–induced transcriptional and proteomic changes in neural cells rather than to examine cancer-specific biology. Nevertheless, we acknowledge that cell line-specific features may influence the observed responses, and future studies will be needed to extend these findings to additional neural and disease-relevant models to further assess their generalizability and translational significance.

Overall, the cognitive effects of ADT are widely overlooked and understudied. Recently, more attention has been drawn to this area with clinical trials such as a Phase II study [32] on cognitive effects of enzalutamide and darolutamide, and another completed Phase 4 trial [50] that found no cognitive changes from AA or enzalutamide during a 12-month follow-up period. Our study underlines the complex molecular changes that take place, revolving around dysfunction in the amyloid pathway, a decrease in key neuroprotective proteins, and impairment in mitochondrial biogenesis that is associated with the presence of androgens.

## 5. Conclusions

Our findings observed in a human neuronal cell model showed the impact of AA on proteins involving in amyloid processing with a consistent significant decrease in APP with AA treatment, a decrease in the neuroprotective proteins synaptophysin and BDNF, and the modulation of mitochondrial biogenesis at different AA dosages. These changes point to reduced neurotrophic support and compromised synaptic integrity. While these data are derived from a neuroblastoma model and not directly linked to clinical cognitive outcomes, they offer a mechanistic framework for future studies to elucidate the pathways driving cognitive decline in patients with advanced prostate cancer. Studying prostate cancer–neuron interactions in co-culture systems offers a controlled platform to interrogate key signaling pathways underlying neuronal health and can illuminate how AA and prostate cancer cells jointly influence neuronal mitochondrial activity.

## Figures and Tables

**Table 1:** Human primer sequences with Tm for real-time PCR.

Primer (Tm)	Forward Sequence	Reverse Sequence
GAPDH (62 °C)	ACCATCATCCCTGCCTCTAC	CCTGTTGCTGTAGCCAAAT
APP (62 °C)	TTTGGCACTGCTCCTGCT	CCACAGAACATGGCAATC
BACE-1 (62 °C)	GCAGGGCTACTACGTGGAGA	CAGCACCCACTGCAAAGTTA
TFAM (63 °C)	AAGATTCCAAGAAGCTAAGGGTGA	CAGAGTCAGACAGATTTTTTCCAGTTT
SYNAPTOPHYSIN (65 °C)	CTGCAATGGGTCTTCGCCA	ACTCTCGGTCTTGTTGGC
ADAM10 (62 °C)	TCGAACCATCACCCTGCAACCT	GCCCACCAATGAGCCACAATCC
NRF-1 (63 °C)	GGCACTGTCTCACTTATCCAGGTT	CAGCCACGGCAGAATAATTCA
BDNF (64 °C)	AGCTATCCAGAGCATCTTCCA	ACCTGGTGGAACTTTATGAAACC
PGC1α (66 °C)	GCATGAGTGTGTGCTCTGTG	GCACACTCGATGTCACTCCA
LRP1 (66 °C)	ATCCAGCTGGACCATAAGGGC	AGGAGAAAGGAACCTACGCCCTC

## Data Availability

The data presented in this study are available upon request from the corresponding author.
